# Genetic variants associated with steatohepatitis and liver fibrosis in HIV-infected patients with NAFLD

**DOI:** 10.3389/fphar.2022.905126

**Published:** 2022-08-30

**Authors:** C. Busca, P. Arias, M. Sánchez-Conde, M. Rico, R. Montejano, L. Martín-Carbonero, E. Valencia, V. Moreno, J. I. Bernardino, A. Olveira, M. Abadía, J. González-García, M. L. Montes

**Affiliations:** ^1^ Unidad VIH, Servicio Medicina Interna, IdiPAz, Hospital Universitario La Paz, Madrid, Spain; ^2^ Instituto de Genética Médica y Molecular (INGEMM), IdiPaz, Hospital Universitario La Paz, Madrid, Spain; ^3^ Infectious Diseases Department, Hospital Universitario Ramón y Cajal, Madrid, Spain; ^4^ Gastroenterology, Hospital La Paz, Madrid, Spain

**Keywords:** HIV, NASH, liver fibrosis, PNPLA3, TM6SF2, MBOAT7-TMC4, liver biopsy, NAFLD

## Abstract

**Background and aims:** Nonalcoholic fatty liver disease (NAFLD) is a common cause of liver damage in people living with HIV (PLWHIV). Several studies have investigated candidate genes for susceptibility to NAFLD and to steatohepatitis. *PNPLA3*, *TM6SF2*, and *MBOAT7-TMC4* have been reported to be associated with elevated ALT levels and the histologic parameters of nonalcoholic steatohepatitis and severity of fibrosis. Our objective was to analyze the relationship between *PNPLA3*, *TM6SF2*, and *MBOAT7-TMC4* and steatosis, steatohepatitis, and liver fibrosis in PLWHIV with NAFLD.

**Method:** A cohort of PLWHIV with persistently elevated aminotransferase levels and suspected NAFLD who underwent liver biopsy and determination of genetic variants was assessed at two large centers in Spain. All participants included in the current study were genotyped for rs738409 (*PNPLA3*), rs58542926 (*TM6SF2*), and rs641738 (*MBOAT7-TMC4*).

**Results:** The study population comprised PLWHIV who were on stable antiretroviral therapy [7.7% women; median age, 49.3 years (44–53.4)]. The median CD4 count was 829 (650–980), 60% had metabolic syndrome, and 18.5% were diabetic. The median BMI was 28.9 (25.5–30.8). Patients with liver steatosis (any grade) vs. nonsteatosis tended to harbor the *PNPLA3* G allele variant [57.6% vs. 16.7% (*p* = 0.09)], but not *TM6SF2* or *MBOAT7-TMC4* variants. However, those with steatohepatitis vs. nonsteatohepatitis significantly more frequently had the *PNPLA3* G allele variant [69.4% vs. 39.1% (*p* < 0.05)] and the *MBOAT7-TMC4* A allele variant [75% vs. 42% (*p* < 0.05)]. In our cohort, the *TM6SF2* gene variant was not associated with steatosis or steatohepatitis. The *PNPLA3* G allele variant was associated with steatohepatitis [OR 4.9 (1.3–18); p 0.02] and liver fibrosis [OR 4.3 (1.1–17.4); p 0.04], and the *MBOAT7-TMC4* A allele variant was associated with steatohepatitis [OR 6.6 (1.6–27.6); p 0.01].

**Conclusion:** The *PNPLA3* G allele variant and *MBOAT7-TMC4* A allele variant were associated with steatohepatitis and liver fibrosis in PLWHIV with persistently elevated aminotransferases and NAFLD. We recommend routine genotyping for *PNPLA3* and *MBOAT7-TMC4* in PLWHIV with NAFLD to identify those at higher risk of progression.

## 1 Introduction

Nonalcoholic fatty liver disease (NAFLD) is increasingly diagnosed in persons living with HIV infection (PLWHIV) and is currently the main cause of liver disease in countries where hepatitis C has been systematically treated and cured in this population (Macías et al., 2014; Sulyok et al., 2015; Lake et al., 2021).

NAFLD is a multifactorial liver disease affected mainly by lifestyle-related variables, such as diet, sedentary lifestyle, and overweight. However, genetic factors are known to play a role in the development and progression of liver disease (Price et al., 2014; Coronel-Castillo et al., 2019). NAFLD has been associated with genetic polymorphisms of enzymes involved in the metabolic pathways of hepatic fatty acids in the general population. The polymorphisms of three genes that encode three proteins are considered to be more clearly associated with this disease, namely, *PNPLA3* (adiponutrin, enzyme involved in triglyceride metabolism), *TM6SF2* (involved in hepatic secretion of very-low density lipoproteins), and *MBOAT7-TMC4* (involved in remodeling of the hepatic phosphatidylinositol acyl chain) (Wood et al., 2015; Mancina et al., 2017).

In addition to metabolic and HIV-related factors and antiretroviral therapy, polymorphisms of these genes and their association with steatosis and liver fibrosis have been studied in PLWHIV with NAFLD and/or viral chronic hepatitis. The results have been contradictory, with few studies including liver biopsy samples. The populations of these studies differed considerably in terms of race, time with HIV infection, coinfection by hepatitis C virus (HCV) (Scheiner et al., 2015; Núñez-Torres et al., 2016), and other comorbid conditions, thus potentially explaining the variability in the results reported (Dold et al., 2017; Sherman et al., 2021). However, to date, no studies have analyzed the association between the genes *PNPLA3*, *TM6SF2*, and *MBOAT7-TMC4* and biopsy-confirmed NAFLD in PLWHIV.

Given that NAFLD is very prevalent in PLWHIV, it is necessary to identify factors associated with a greater risk of progression to nonalcoholic steatohepatitis (NASH), such as liver fibrosis, in order to target and follow this disease in the highest-risk patients (Krahn et al., 2020; Lake et al., 2021). The objective of this study was to determine the association between variants of *PNPLA3, TM6SF2,* and *MBOAT7-TMC4* and the three histologic stages of NAFLD (steatosis, NASH, and fibrosis) in PLWHIV.

## 2 Patients and methods

### 2.1 Study cohort

The study population comprised PLWH followed at Hospital Universitario La Paz, Madrid, Spain and Hospital Universitario Ramón y Cajal, Madrid, Spain from January 2017 to June 2018. All patients had increased transaminase levels for ≥6 months (confirmed by ≥ 2 samples) and suspected NAFLD and agreed to undergo a liver biopsy to complete their clinical assessment. Of the 65 patients included in the study and who underwent liver biopsy, we excluded other hepatic causes of persistently elevated transaminases, as follows: viral infections, high consumption of alcohol and recreational drugs, medication, and congenital or autoimmune liver disease. The biopsies were performed to complete the diagnosis of increased transaminases. Other inclusion criteria were being on stable antiretroviral therapy and having HIV-RNA <50 copies/ml for ≥1 year. The exclusion criteria were past or present chronic HBV or active HCV coinfection, high alcohol consumption (>30 g/d in men or ≥20 g/d in women), potential drug-induced hepatotoxicity, and other liver diseases.

Participants were enrolled in the study after providing their written informed consent. The study was approved by the local ethics committee (code PI-2248) and conducted according to the Declaration of Helsinki.

All patients underwent screening for cardiovascular risk factors (NCEP, 2001) and autoimmune, genetic, and metabolic liver disease, as well as liver ultrasound and measurement of liver stiffness and steatosis by transient elastography (TE) and controlled attenuation parameter (CAP) (FibroScan Echosens Paris^®^). Liver biopsy was offered to all patients according to the EASL-EASD guidelines. (Marchesini et al., 2016). All patients agreed to undergo biopsy.

### 2.2 Determination of single-nucleotide polymorphisms

Genomic DNA was extracted from peripheral blood cells using the Chemagen chemagic kit (Perkin Elmer, United States) following the manufacturer’s instructions.

Single-nucleotide polymorphisms (SNPs) in *PNPLA3*, *TM6SF2*, and *MBOAT7-TMC4* were analyzed using Sanger sequencing, as follows (primers available upon request):

- P*NPLA3* rs738409 on chromosome 20. The three genotypes of rs738409 detected were CC, CG, and GG. The C genotype is considered the wild-type allele and the G genotype the allele of interest.
- T*M6SF2* rs58542926 on chromosome 19. The three genotypes of rs58542926 detected were GG, GA, and AA. The G genotype is considered the wild-type allele and the A genotype the allele of interest.
- M*BOAT7-TMC4* rs641738 on chromosome 19. The three genotypes of rs641738 detected were GG, GA, and AA. The G genotype is considered the wild-type allele, because it is the most frequent in our population, and the A genotype the allele of interest.

### 2.3 Liver biopsy

Ultrasound-guided percutaneous liver biopsy was performed with a 16G needle. Liver histology was interpreted by two experienced liver pathologists, who were blind to clinical data (including noninvasive test results). Only liver biopsies with >11 portal spaces were considered valid for the study.

Liver steatosis and steatohepatitis (NASH) [assessed using the NAFLD activity score (NAS)] were categorized using the scoring system proposed by Kleiner et al., i.e., a NAS of ≥3 correlated with a diagnosis of NASH (presence of at least 5% steatosis with at least both grade 1 hepatocellular ballooning and lobular inflammation following a mainly centrilobular distribution). Biopsies with scores of <3 were diagnosed as “not NASH.” Liver fibrosis was classified using the METAVIR system, with mild fibrosis defined as F0–F2 and advanced fibrosis as F3–F4 (i.e., bridging fibrosis and cirrhosis) (Kleiner et al., 2005).

### 2.4 Liver ultrasound

Liver ultrasound was carried out by a single person with broad experience in performing and interpreting hepatic ultrasound. The technical parameters (gain adjustment, focal zone placement, and optimum placement of the transducer) were tailored to the individual patient. Presence or absence of hepatic steatosis was based on the overall impression using the ultrasound abnormalities detected. The findings that were specifically evaluated included hepatorenal echo contrast, bright liver echo, deep attenuation, vessel blurring, and non-specific findings of heterogeneous echoes (Kromrey et al., 2019).

### 2.5 Transient elastography

TE was performed under fasting conditions using a FibroScan device (Probe M, FS402; Echosens, France), with measurement of CAP. The cut-off value for diagnosis of steatosis was >238 dB/m (Sasso et al., 2012). An experienced operator, who was blind to the liver ultrasound diagnosis, performed TE following the manufacturer’s protocol, according to which F ≥ 2 was defined as a TE value ≥ 7.0 kPa and F ≥ 3 (advanced) as a TE value > 9.6 kPa (Wong et al., 2010).

### 2.6 Statistical analysis

Categorical variables were expressed as proportions; continuous variables were expressed as median and interquartile range (IQR). Continuous variables were analyzed using the *t* test or Mann-Whitney test, depending on the normality of the distribution. Categorical variables were compared using the chi-square or the Fisher exact test, as appropriate.

The impact of genetic markers on NASH and liver fibrosis was analyzed using logistic regression. Variables with a *p* value < 0.1 and/or that were clinically relevant were included in the multivariable models. Nevertheless, some clinical variables that we considered sufficiently important for fitting the regression models were included with a *p* value < 0.2. Furthermore, we performed a regression analysis using a Markov chain Monte Carlo–type model for imputation of missing data. The analysis was iterative to ensure the validity of the results, with all study participants included.

Data were analyzed using SPSS for Windows, Version 26.0 (IBM Corp., Armonk, NY, United States). Statistical significance was set at *p* < 0.05 (2-tailed) for all tests.

## 3 Results

### 3.1 Study population

A total of 65 patients underwent liver biopsy and genetic determinations [92% men, 88% White, median age 49 (44–53) years]. The study population had a long history of HIV infection, which was well-controlled [median time with HIV infection, 14 (7–21) years]. At baseline HIV viral load was undetectable in all cases (100%). The median CD4 cell count was 829 (650–980) cells/mm^3^ (Table 1).

**TABLE 1:** Characteristics of the study population.

		Patients included (N = 65)
Age (years)^a^		49.3 (44–53.4)
Female sex, N (%)		5 (7.7)
Ethnicity, N (%)	White	53 (88.3)
	Black	2 (3.3)
	Asian	0 (0)
	Latin American	5 (8.3)
Stage C AIDS, N (%)		11 (20.8)
Route of transmission, N (%)	MSM	42 (64.6)
	MSW	11 (16.9)
	IDU	5 (7.7)
	Transfusion/Hemophilia	3 (4.6)
	Unknown	2 (3.1)
HIV-viral load <50 copies/ml, N (%)		65 (100)
Time with HIV infection (years)^a^		14.3 (7.4–20.6)
Nadir CD4 cell count (/µl)^a^		294 (187–438)
CD4 cell count (/µl)^a^		828.9 (650–980)
ART, N (%)	2 NRTI + RPV/2 NRTI + EFV	24 (36.9)/6 (9)
	2 NRTI + INI	19 (29.2)
	PI-based regimen	9 (13.8)
	Other	7 (11)
BMI (kg/m^2^)^a^		28.9 (25.5–30.8)
Waist circumference (cm)		101 (92–110)
AST (IU/L)^a^		36 (29–47)
ALT (IU/L)^a^		54 (42–83)
GGT (IU/L)^a^		46 (30–101)
Glucose (mg/dl)^a^		103 (96–110)
Cholesterol (mg/dl)^a^		179 (157–202)
LDL-C (mg/dl)^a^		108 (92–124)
HDL-C (mg/dl)^a^		38 (34–45)
Triglycerides (mg/dl)^a^		158 (110–227)
Diabetes mellitus or abnormal fasting glucose, N (%)		43 (66.2)
Arterial hypertension, N (%)		37 (56.9)
Dyslipidemia, N (%)	Hypercholesterolemia, N (%)	31 (47.7)
	Hypertriglyceridemia, N (%)	38 (58.5)
	Mixed, N (%)	31 (47.7)
Cardiovascular events, N (%)^b^		1 (1.5)
Metabolic syndrome, N (%)		39 (60)
Lipid-lowering drugs, N (%)		27 (41.5)
Glucose-lowering drugs, N (%)	Metformin	16 (24.6)
	Insulin	0 (0)

a Median (IQR).

b Cardiovascular events: ischemic heart disease and ischemic stroke.

PI-based regimen: any combination which included a protease inhibitor.

INI, integrase inhibitor; RPV, rilpivirine; EFV, efavirenz.

MSM, men sex men; MSW, mens sex women; IDU, intravenous drug users.

Regarding the metabolic characteristics of the cohort, median BMI was 29 (25–31), and 60% had metabolic syndrome. A total of 66% of patients had diabetes or impaired fasting glucose, and 48% had dyslipidemia (hypercholesterolemia, hypertriglyceridemia, or both) (Table 1).

We analyzed patient characteristics after subdividing the study population into two groups according to the polymorphisms evaluated (Table 2). As for *PNPLA3*, no differences were observed for any variables, except presence of steatohepatitis and fibrosis. In the case of *MBOAT7*, differences were recorded for sex, with the polymorphism of interest less frequent in women. Differences were also observed for presence of arterial hypertension, abnormal fasting glucose, and steatohepatitis.

**TABLE 2:** Patient characteristics according to polymorphisms in *PNPLA3* and *MBOAT7-TMA*.

	PNPLA3 (N = 65)			MBOAT7-TMC4 (N = 56)		
	CC (N = 30)	CG or GG (N = 35)	*p*	GG (N = 19)	GA or AA (N = 37)	*p*
Age (years)^a^	49.3 (42.1–52.9)	49.9 (44–53.8)	0.742	47.8 (42.1–53.1)	51.6 (47.3–55.1)	0.092
Female sex, N (%)	3 (10)	2 (5.7)	0.655	4 (21.1)	1 (2.7)	**0.041**
Stage C AIDS N (%)	6 (26.1)	5 (16.7)	0.501	2 (12.5)	8 (26.7)	0.455
Nadir CD4 cell count, (/µl)^a^	326 (170–441)	260 (190–400)	0.740	254 (182–375)	326 (190–465)	0.393
CD4 cell count (/µl)^a^	867.2 (710.5–980)	816 (603.1–999)	0.155	842.8 (611.2–963)	850 (651.6–1020)	0.640
HIV infection time (years)^a^	11.7 (6.4–18.3)	17.9 (8.4–20.9)	0.242	14.7 (5.6–24.7)	14.3 (7.7–19.2)	0.528
BMI (kg/m^2^)^a^	26.7 (24.4–30.8)	29.6 (27–31.3)	0.106	29.3 (25.4–31.3)	28.9 (25.2–30.8)	0.723
AST (IU/L)^a^	32.5 (29–43)	37 (30–49)	0.197	33 (27–42)	39 (30–49)	0.250
ALT (IU/L)^a^	51.5 (41–77)	61 (42–87)	0.190	45 (36–79)	60 (44–84)	0.106
GGT (IU/L)^a^	37 (29–87)	52 (32–101)	0.452	43 (29–85)	50 (32–118)	0.458
Glucose (mg/dl)^a^	104 (96–107)	101 (94–115)	0.693	101 (90–112)	105 (99–110)	0.267
Cholesterol (mg/dl)^a^	179.5 (156–202)	172 (157–207)	0.727	169 (153–192)	180 (162–207)	0.171
LDL-C (mg/dl)^a^	107.5 (96–124)	108 (87–124)	0.400	96 (86–122)	112 (92–126)	0.287
HDL-C (mg/dl)^a^	38.5 (34–46)	37 (33–44)	0.493	38 (30–44)	37 (34–45)	0.579
Triglycerides (mg/dl)^a^	157 (109–234)	158 (110–227)	0.767	155 (97–226)	160 (125–231)	0.505
APRI score^a^	0.3 (0.3–0.5)	0.5 (0.3–0.6)	0.060	0.4 (0.3–0.5)	0.4 (0.3–0.6)	0.815
FIB4 score^a^	0.9 (0.7–1.1)	1.1 (0.9–1.5)	0.055	0.9 (0.8–1.2)	1 (0.7–1.3)	0.952
TyG score^a^	9 (8.6–9.3)	9 (8.6–9.4)	1.000	9.1 (8.5–9.2)	9.1 (8.8–9.4)	0.462
Arterial hypertension, N (%)	16 (53.3)	21 (60.0)	0.623	6 (31.6)	26 (70.3)	**0.010**
Diabetes mellitus, N (%)						
No	13 (43.3)	9 (25.7)		7 (36.8)	10 (27.0)	
Yes	4 (13.3)	8 (22.9)		7 (36.8)	5 (13.5)	
AFG	13 (43.3)	18 (51.4)	0.287	5 (26.3)	22 (59.5)	**0.040**
Glucose-lowering drugs, N (%)	9 (30)	16 (45.7)	0.213	9 (47.4)	14 (37.8)	0.572
Dyslipidemia, N (%)						
HCT	16 (53.3)	15 (42.9)	0.460	7 (36.8)	20 (54.1)	0.267
HTG	17 (56.7)	21 (60.0)	0.806	10 (52.6)	24 (64.9)	0.401
Mixed	16 (53.3)	15 (42.9)	0.460	7 (36.8)	20 (54.1)	0.267
Lipid-lowering drugs, N (%)	13 (43.3)	14 (40.0)	0.806	8 (42.1)	16 (43.2)	1.000
Metabolic syndrome	17 (56.7)	22 (62.9)	0.623	13 (68.4)	23 (62.2)	0.772
Transient elastography (kPa)^a^	5.3 (4.3–6.4)	6.85 (4.1–9)	0.166	6 (4.1–7.6)	6.5 (4.8–9.6)	0.326
CAP (dB/m)^a^	306 (256–345)	302 (257–353)	0.789	305 (243–362)	301 (248–353)	0.480
Liver Biopsy, N (%)						
Steatosis	25 (83.3)	34 (97.1)	0.087	19 (100.0)	32 (86.5)	0.155
Steatohepatitis	11 (44)	25 (73.5)	**0.031**	8 (42.1)	24 (75.0)	**0.035**
Fibrosis ≥ F1	4 (13.3)	15 (42.9)	**0.013**	7 (36.8)	10 (27.0)	0.543
Fibrosis ≥ F3	1 (3.3)	2 (5.7)	1.000	0 (0.0)	3 (8.1)	0.544

a Median (IQR).

Allele subtypes and number of subject with them.

Liver steatosis was identified in 88% and 82% of patients by liver ultrasound and CAP, respectively, and in 94% using blood biomarkers. Liver stiffness (measured using TE) compatible with advanced liver fibrosis (F3–F4) was identified in 4.6% of patients.

### 3.2 Histopathological findings

Liver steatosis affected 59 of the 65 patients who underwent liver biopsy (90.8%) [mild, 31 (52.5%); moderate, 15 (25.4%); severe, 13 (22%)]. NASH was diagnosed in 36 cases (61%). A NAS ≥5 was found in 21.5% of patients with NASH; 29.2% had liver fibrosis [mild (F1–F2), 24.6%; severe (>F3), 4.6%]. In the six patients in whom steatosis was not recorded the diagnoses were cryptogenic hepatitis (one patient) and no abnormalities (five patients).

### 3.3 Genetic variants

#### 3.3.1 Distribution of PNPLA3 genotypes

**FIGURE 1:**
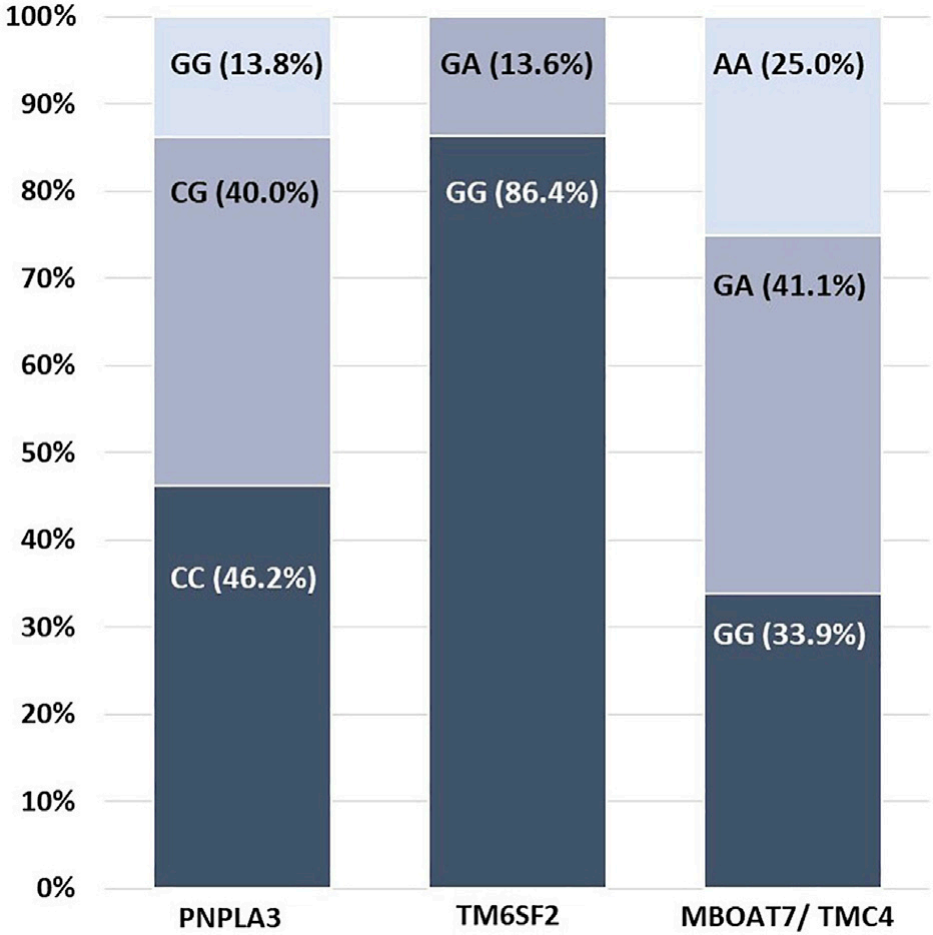
Distribution of allele variants for each gene studied.

The distribution of SNPs in *PNPLA3* (rs738409) was determined. CC (wild-type) was present in 30 participants (46.2%), of whom 40% were heterozygous for CG. Nine patients (13.8%) were homozygous for GG (Figure 1).

#### 3.3.2 Distribution of TM6SF2 genotypes

The distribution of SNPs in *TM6SF* (rs58542926) was 86.4% for GG (wild-type) and 13.6% for heterozygous GA. No cases of homozygous AA variant were detected (Figure 1).

#### 3.3.3 Distribution of MBOAT7 genotypes

The *MBOAT7-TMC4* rs641738 GA genotype was the most common in the cohort (41.1%), followed by GG (33.9%) and AA (25%). This distribution was in Hardy–Weinberg equilibrium (Figure 1).

### 3.4 Association between genetic factors and nonalcoholic fatty liver disease

**FIGURE 2:**
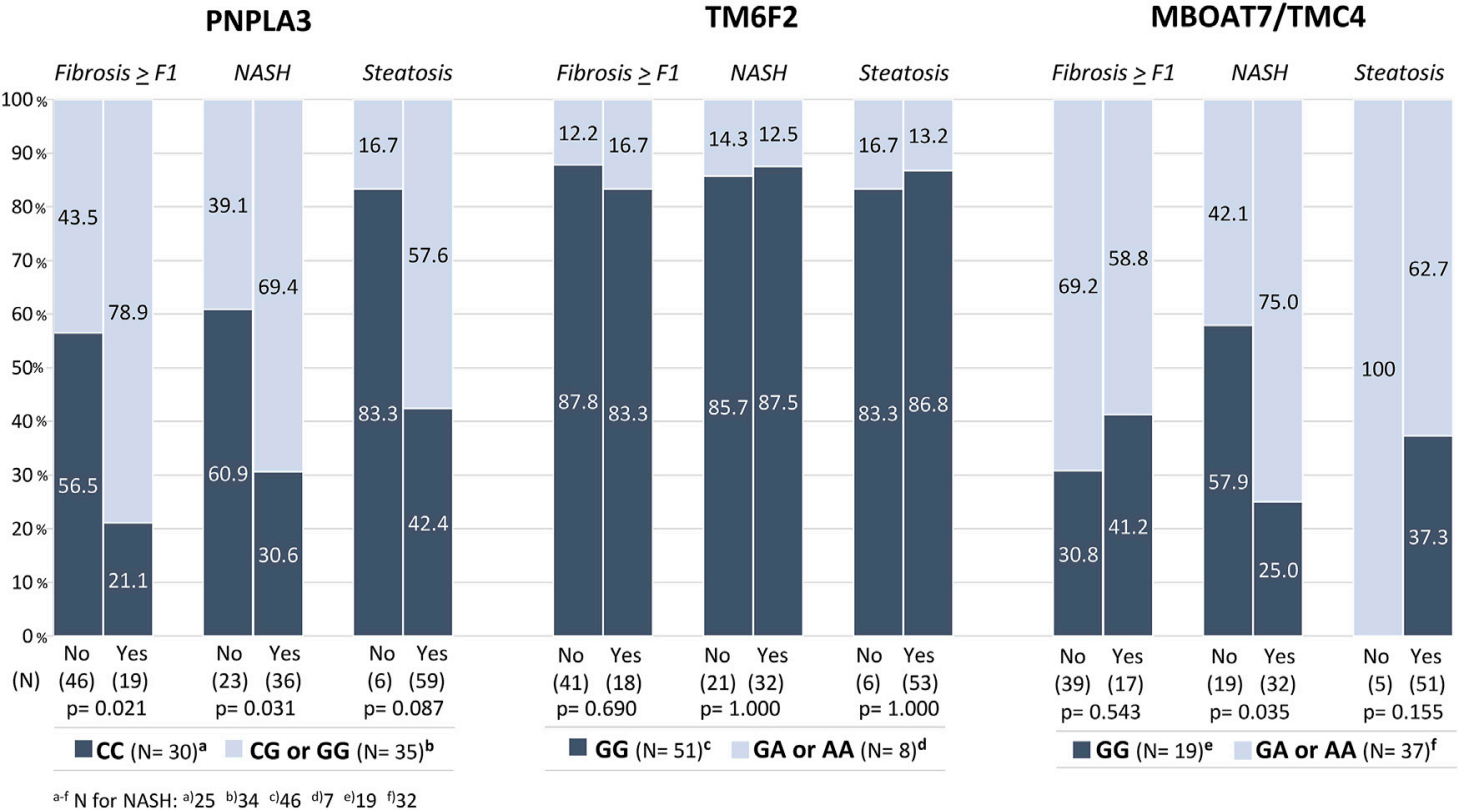
**(A–C)**: Distribution of allele variants in each histopathological category.

We observed that patients with steatosis (any grade) more frequently harbored the *PNPLA3* G allele variant [57.6% vs. 16.7% (*p* = 0.09)] than nonsteatosis patients. However, no differences were found in the proportion of patients with variants of interest in the genes coding for *TM6SF2* (A allele) and *MBOAT7-TMC4* (A allele). In contrast, patients with NASH significantly more frequently harbored the *PNPLA3* G allele variant [69.4% vs. 39.1% (*p* = 0.03)] and *MBOAT7-TMC4* A allele variant [75% vs. 42.1% (*p* = 0.03)]. The *TM6SF2* A allele variant was not associated with steatosis or NASH. As for liver fibrosis ≥ F1, the *PNPLA3* G allele variant was significantly more frequent (78.9% vs. 43.5%; *p* = 0.01) Figures 2A–C shows the distribution of SNPs in the study population.

We found the factors independently associated with NASH to be body mass index and harboring the *PNPLA3* G variant and the *MBOAT7-TMC4* A variant. Differences for variables associated with insulin resistance, presence of DM, and glycemia were close to significance. In the multivariable model that included glucose instead of DM or abnormal fasting glucose, the difference was very close to significance (*p* = 0.051). We observed that harboring the *PNPLA3* G variant and aspartate aminotransferase levels were independently associated with liver fibrosis (any grade). No multivariable analysis was performed for factors associated with steatosis owing to the low number of disease-free patients in the biopsies studied (Table 3).

**TABLE 3:** Multivariate analysis for liver fibrosis and steatohepatitis.

	Fibrosis		NASH	
	Univariable	Multivariable (stepwise)	Univariable	Multivariable (stepwise)
Age (years)	1 (1–1.1); 0.273^a^		1 (1–1.1); 0.503^a^	
Sex	0.6 (0.1–5.6); 0.64		0.4 (0.1–2.6); 0.327	
HIV infection time (years)	1 (1–1.1); 0.202^a^	**1.1 (1–1.2); 0.101**	1 (1–1.1); 0.686^a^	
CD4 cell count (/µL)	1 (1–1); 0.103^a^		1 (1–1); 0.98	
BMI (kg/m^2^)	1.1 (0.9–1.2); 0.325^a^		1.2 (1–1.4); 0.024^a^	**1.2 (1–1.4); 0.037**
AST (IU/L)	1.1 (1–1.1); 0.005^a^	**1.1 (1–1.1); 0.008**	1 (1–1.1); 0.141	
ALT (IU/L)	1 (1–1); 0.033^a^		1 (1–1); 0.106	
Glucose (mg/dl)	1 (1–1.1); 0.043^a^		1.1 (1–1.1); 0.015^a^	**1.1 (1.0–1.1); 0.051**
Cholesterol (mg/dl)	1 (1–1); 0.679		1 (1–1); 0.59	
Triglycerides (mg/dl)	1 (1–1); 0.435		1 (1–1); 0.248	
DM or abnormal fasting glucose	3.8 (1–14.7); 0.058		2.7 (0.9–8.4); 0.088	
Metabolic syndrome	1.2 (0.4–3.6); 0.739		1.5 (0.5–4.5); 0.433	
PNPLA3 allele G	4.9 (1.4–17); 0.013^a^	**4.7 (1.2–18.8); 0.027**	3.5 (1.2–10.6); 0.024^a^	**5.1 (1.3–20.5); 0.023**
TM6SF2 allele A	1.4 (0.3–6.8); 0.645		0.9 (0.2–4.3); 0.851	
MBOAT7-TMC4 allele A	0.6 (0.2–2.1); 0.451		4.1 (1.2–13.9); 0.022^a^	**6.0 (1.3–27.6); 0.022**

a Variables included in the multivariable stepwise analysis. OR (95%CI); *p*.

Dependent variable of the analysis (Fibrosis(NASH) and type of analysis performed.

## 4 Discussion

The results of our study show the association between genetic polymorphisms in *PNPLA3* and the presence of biopsy-confirmed NAFLD in PLWHIV. The G allele variant was more common in patients with steatosis and liver fibrosis. We also demonstrated, for the first time in PLWHIV, a significant association between the *MBOAT7-TMC4* A variant and steatohepatitis. The absence of an association between *TM6SF2* variants and NAFLD could be because of the low number of A allele variants.

The first studies to analyze associations between the severity of chronic liver disease and progression of fibrosis and allelic variants in *PNPLA3* were performed in the context of HCV coinfection in PLWHIV (Scheiner et al., 2015; Núñez-Torres et al., 2016; Dold et al., 2017; Franco et al., 2021). All the studies found results pointing to this association, and it is now widely accepted that the presence of the G allele variant in *PNPLA3* increases the risk of progression to advanced fibrosis by two- to 3-fold.

Studies analyzing the association between *PNPLA3* and NAFLD in PLWHIV are more recent and report varying results (Sherman et al., 2021) (Price et al., 2014) (Dold et al., 2017). It is important to note that the diagnostic methods used to identify NAFLD included CAP, liver ultrasound, and magnetic resonance, thus explaining in part this variability. Such variable results could also be explained by racial and ethnic differences between the populations in the various studies. It is well known that problem alleles are not very frequent in Black individuals, whereas Latin Americans more frequently have the G allele (Sherman et al., 2021). The percentages found for the different allelic variants of each gene in our study are within the expected frequencies in study populations according to race and ethnicity, thus explaining why we did not find an AA homozygote in *TM6SF2* (the expected frequency is 0.08 in Europeans and 0.03 in Latin Americans) (Trépo and Valenti, 2020). The G variant in *PNPLA3* was recorded in 53% of cases, thus demonstrating that *PNPLA3* I148M accounts for more than half of the interethnic variability in predisposition to liver disease, especially steatosis, steatohepatitis, and fibrosis of varying origin (viral, alcohol, or metabolic) (Karlsen, 2009). Our study population was more homogeneous than that of other studies and comprised mainly White males in whom the route of transmission of HIV was sexual relations between men who have sex with men; we believe this gives consistency to our results in this subgroup of PLWHIV in our setting and underscores the interest in targeting *PNPLA3* and *MBOAT7* in mainly White and Latin American populations.

We found that being a carrier of the G variant of *PNPLA3* increased 5-fold the probability of NASH and 4-fold the probability of liver fibrosis. These results enable us to identify a population in which every effort must be made to treat NAFLD in order to prevent progression to advanced liver disease and more closely monitor liver damage in affected patients. Additionally, a novel aspect of our study was the analysis of variants of *MBOAT7* in PLWHIV, which showed that carriers of the A variant are 6 times more likely to have NASH. In fact, we observed that patients carrying the A variant more frequently have arterial hypertension and impaired fasting glucose, which are the components of metabolic syndrome that most commonly affect the liver in steatohepatitis (Xia et al., 2021) (Teo et al., 2021). We believe that, given the lack of good noninvasive markers of NASH, any tool that helps to identify patients with NASH without necessitating biopsy is extremely interesting and applicable. Moreover, given the clear need to find specific treatments to reverse both NASH and liver fibrosis in the general population and in PLWHIV, we believe that genetic variants also have a role to play in the more rapid and efficient selection of patients for inclusion in clinical trials on treatment of NAFLD. Recent studies have begun to analyze the impact of various strategies for treatment of NAFLD and their response according to the presence of the allelic variants of the genes we evaluated. The results are very varied, although they point to a new usefulness of genetic studies for the design of tailored treatment strategies in the coming years (Dallio et al., 2021). Other groups are exploring the association between variants in *PNPLA3-TM6SF2-GCKR-MBOAT7* in order to design polygenic risk scores (PRS) that can be evaluated in the clinic to gain insight into the causal relationship between NAFLD and hepatocellular carcinoma (HCC) and to stratify risk of HCC. Variants in *PNPLA3-TM6SF2-GCKR-MBOAT7* were combined in a hepatic fat content PRS (PRS-HFC) (Bianco et al., 2021). In our opinion, the appearance of lines of research that include the allelic variants of the genes we evaluated demonstrates the considerable current interest in this field and the huge potential for development and implementation of this research in the near future. Our findings for PLWHIV with a higher risk of NAFLD, progression of liver fibrosis, and HCC than in the general population highlight the clear interest of our study.

Our study is subject to the limitations inherent to small studies. Nevertheless, it is important to remember that the size of our sample resulted from the difficulty obtaining biopsy specimens to evaluate liver damage. Equally noteworthy are the fact that our results cannot be extended to other populations, especially HIV-infected women (around 20% of PLWHIV in our setting), and the lack of a control group to compare the results. In addition, in our study, only 4% of patients had advanced fibrosis (F3, F4), thus preventing us from performing a specific analysis of this subpopulation. Nevertheless, previous studies on the allelic variants of *PNPLA3* demonstrate their association with the different stages of liver fibrosis. It seems that *PNPLA3* mutant protein induces liver fibrosis by acting on hepatic stellate cells and not on other fibrogenic cells involved in later stages of fibrosis, thus explaining the interest in studying patients in whom the mechanisms of fibrosis are already activated, irrespective of disease stage (Pingitore et al., 2016). It would have been interesting to have a control group of patients with steatohepatitis who were not HIV-infected, unfortunately we were unable to include patients with these characteristics. Nevertheless, our results for the association between CG/GG in PNPLA3 and presence of steatohepatitis and fibrosis in the general population are well documented (Krawczyk et al., 2017; Mazo et al., 2019; Paternostro et al., 2021) and the main interest of our study was that we explored whether findings previously reported in other populations can also be found in populations coinfected by HIV without coinfection by HCV. Our results also show an independent association between CG/GG in PNPLA3 and the presence of steatohepatitis and liver fibrosis in this population.

More prospective studies with a larger number of both sexes are necessary to analyze the impact of these genetic factors on the course of NAFLD in PLWHIV and to determine whether available treatments for **N**AFLD in this population provide the same benefits according to the allelic variants of *PNPLA3* and *MBOAT7-TMC4* in an individual patient.

In conclusion, our results indicate that genotyping of *PNPLA3* rs738409 and *MBOAT7-TMC4* rs641738 could help to identify PLWHIV with NAFLD, NASH, and liver fibrosis and to optimize follow-up and treatment of diseases associated with NAFLD. This approach could also be used to select candidates for clinical trials on the pharmacologic treatment of NASH. We suggest routine genotyping for *PNPLA3* and *MBOAT7-TMC4* in White and Latin-American PLWHIV with NAFLD.

## Data Availability

The original contributions presented in the study are included in the article/Supplementary Materials, further inquiries can be directed to the corresponding author.
